# Supplementary material for: Synthesis of an aromatic N-heterocycle derived from biomass and its use as a polymer feedstock
Source: Nat Commun. 2019 May 8;10:2107. doi: 10.1038/s41467-019-10178-0 (PMC6506500; doi:10.1038/s41467-019-10178-0)
Supplement: Supplementary file 1 — Supplementary Information [file 41467_2019_10178_MOESM1_ESM.pdf]

Supplementary Information

Synthesis of an aromatic N-heterocycles derived from biomass and its use as a polymer feedstock

Qi et al.

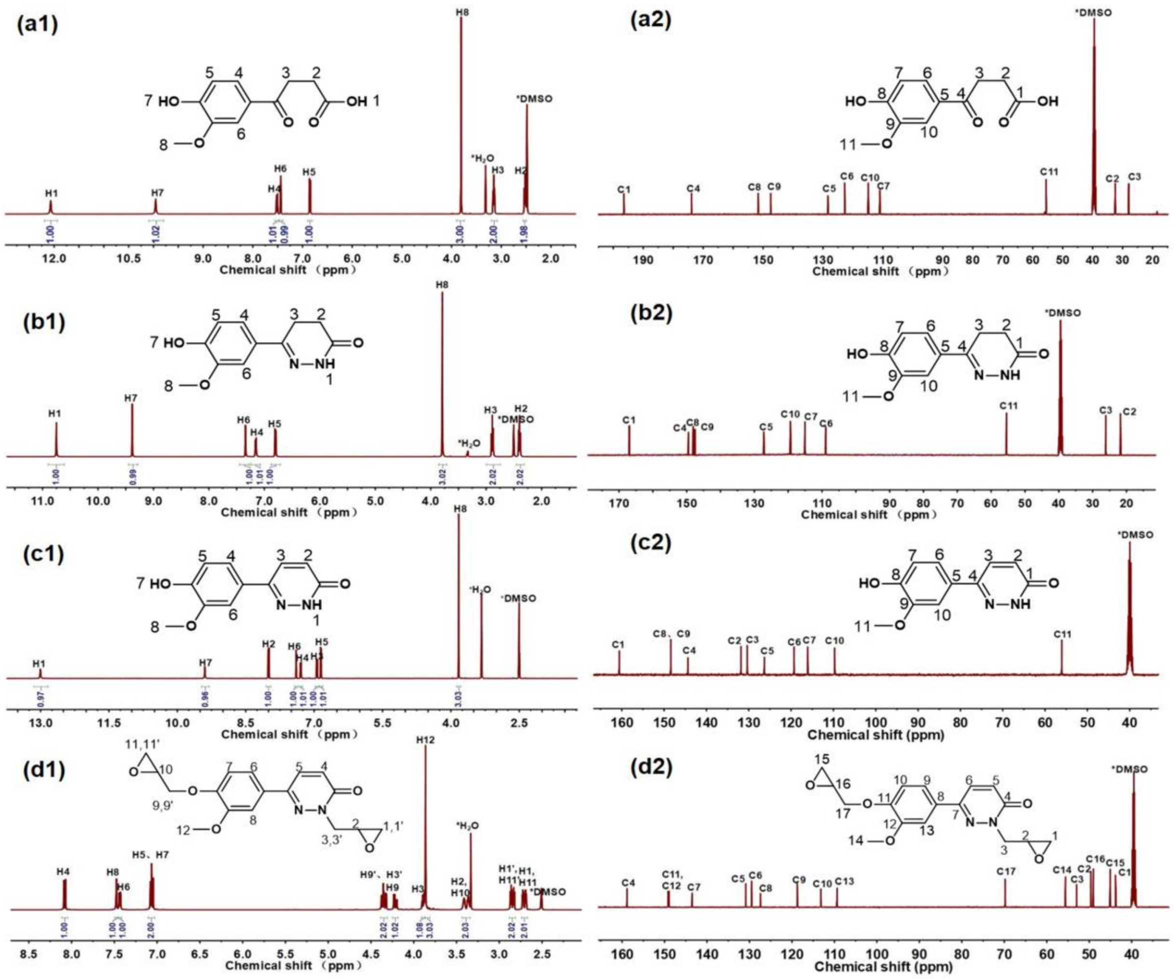

Supplementary Figure 1 The NMR characterization of biobased monomers. ^{1}H-NMR spectra of (a1) GSA, (b1) GSHZ, (c1) GSPZ, (d1) GSPZ-EP. ^{13}C-NMR spectra of (a2) GSA, (b2) GSHZ, (c2) GSPZ, (d2) GSPZ-EP.

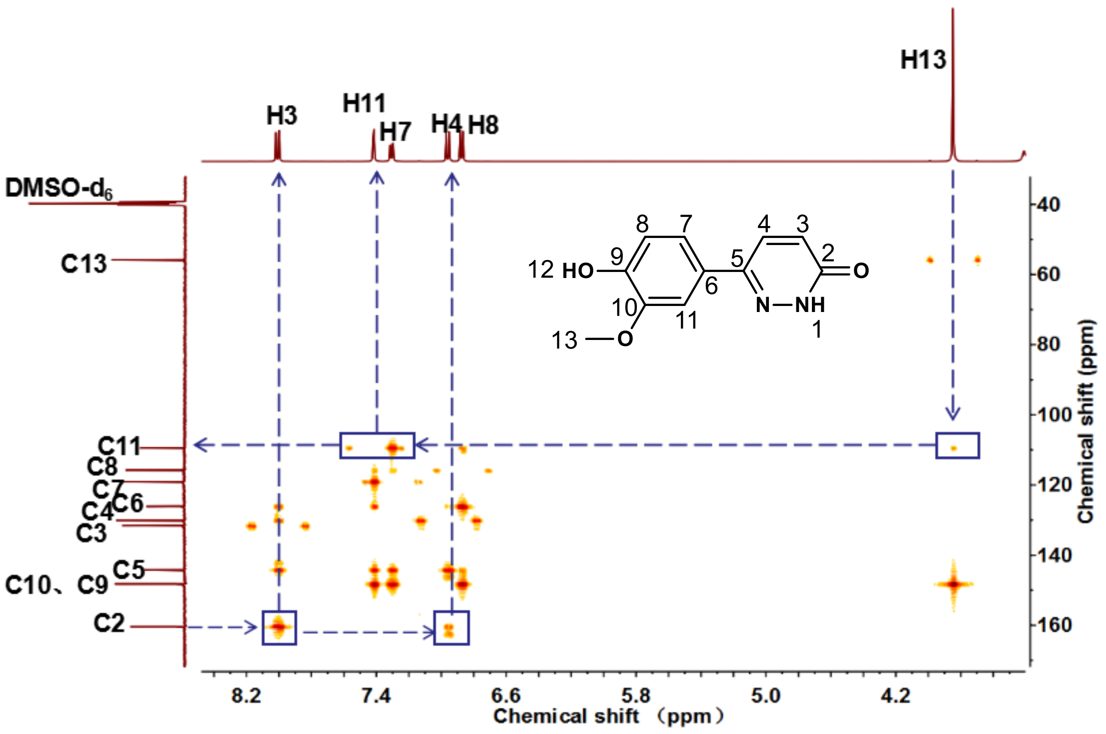

Supplementary Figure 2 HMBC spectrum of GSPZ.

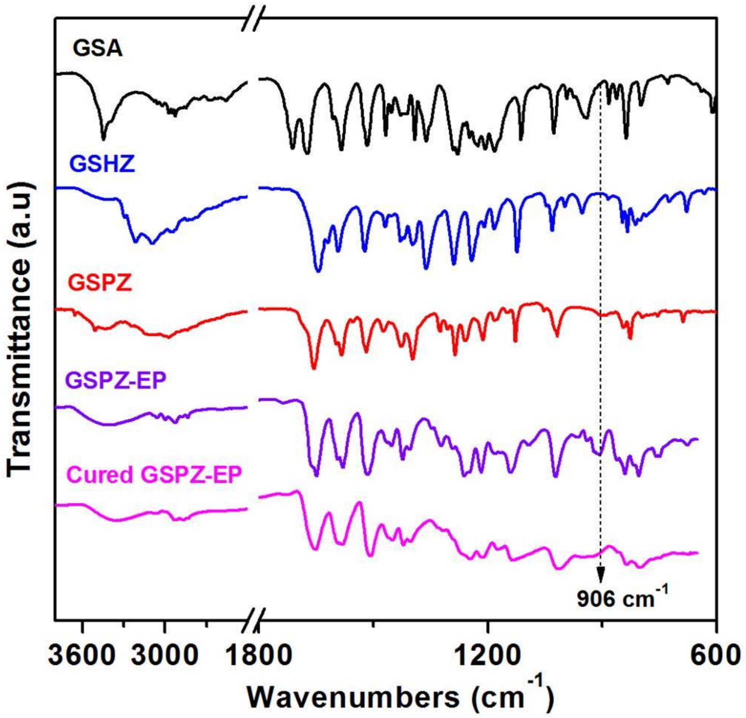

Supplementary Figure 3 FT-IR spectra of GSA, GSHZ, GSPZ, GSPZ-EP and cured GSPZ-EP.

The characteristic peaks of 3450 cm^{-1} and 1712 cm^{-1} are attributed to O-H and C=O of carboxyl acid group of GSA respectively. While for GSPZ these peaks disappeared, and the broad peak of 3300-3500 cm^{-1} attributed to O-H and N-H. In addition, characteristic peak of 1658 cm^{-1} ascribed to C=O of amide group appear. For GSPZ-EP, the characteristic peak of epoxy group at about 906 cm^{-1} appear. while for the cured GSPZ-EP/DDM, the epoxy characteristic peak of 906 cm^{-1} disappeared completely, which indicated the GSPZ-EP could cured completely under the selected curing conditions.

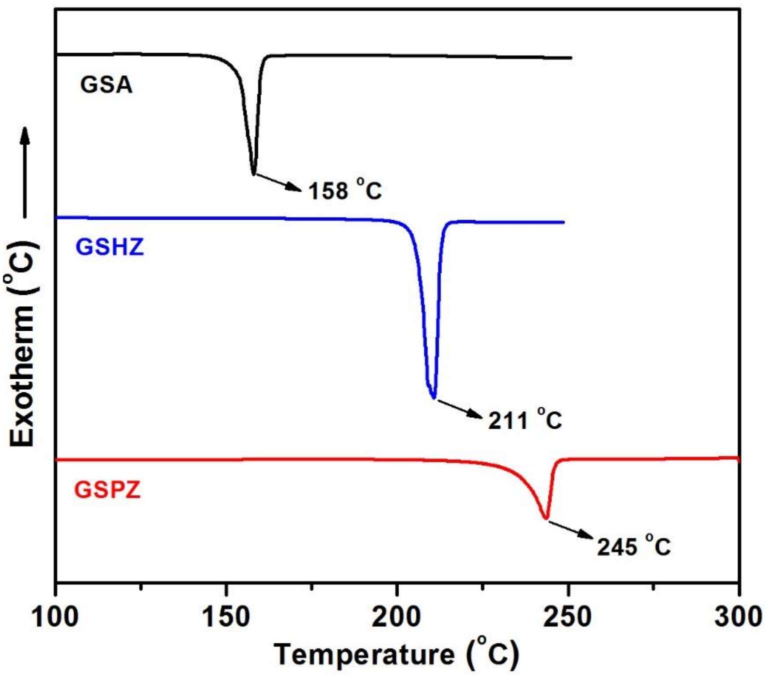

Supplementary Figure 4 Melting point of biobased compounds. DSC curves of GSA, GSHZ and GSPZ to detect the melting point of these compounds.

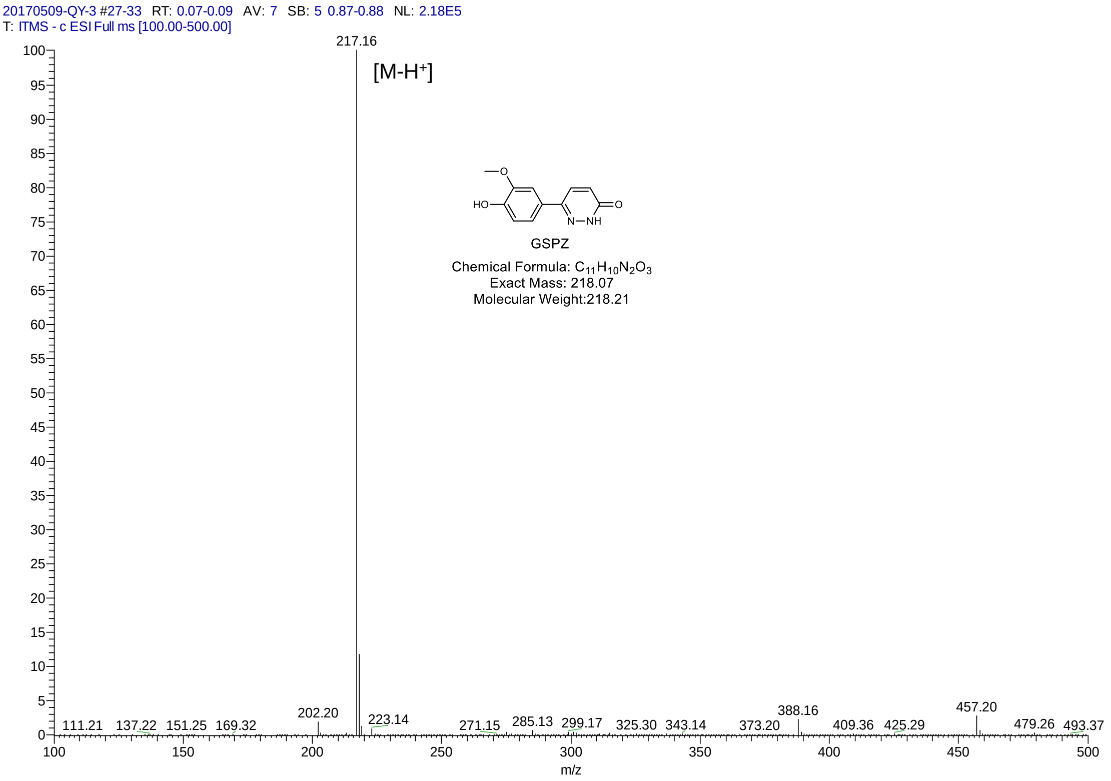

Supplementary Figure 5 ESI-MS spectrum of GSPZ.

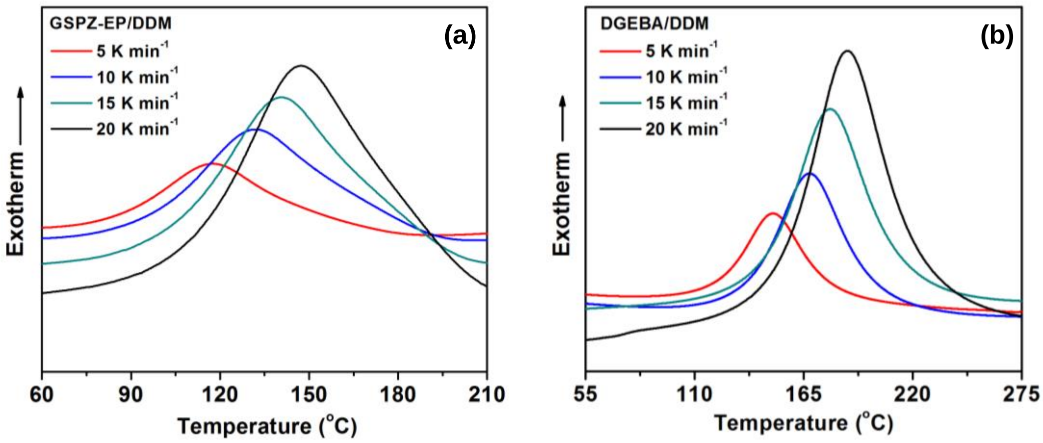

Supplementary Figure 6 Curing kinetics characterization of GSPZ-EP/DDM and DGEBA/DDM. Curing kinetic curves for heat flow against temperature with heating rates of 5,10,15,20K·min^{-1}. (a) bio-based GSPZ-EP/DDM system; (b) petroleum-based DGEBA/DDM system.

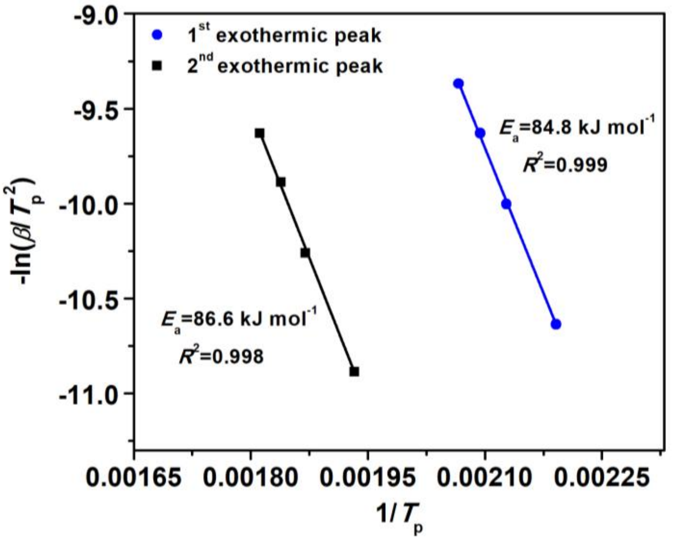

Supplementary Figure 7 The curing apparent activation energy of self-curing GSPZ-EP. Linear fit plots of ln(β/T_p^2) vs 1/T_p based on Kissinger's equation for the 1st and the 2nd exothermic peaks of GSPZ-EP.

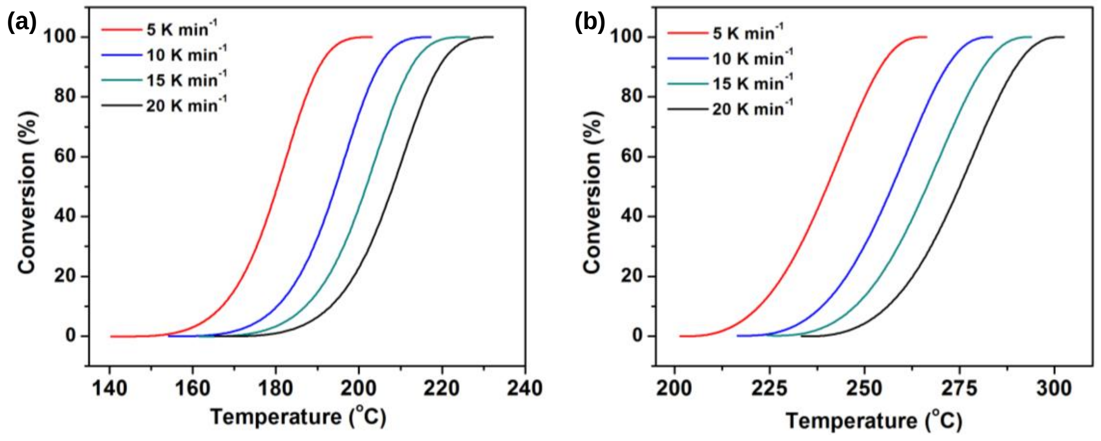

Supplementary Figure 8 Conversion of GSPZ-EP as a function of temperature for (a) the 1st exothermic peak of GSPZ-EP; (b) the 2nd exothermic peak of GSPZ-EP.

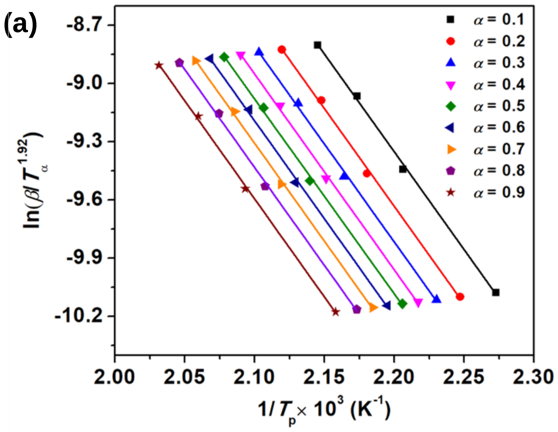

Supplementary Figure 9 Starink plots at various degrees of conversation for (a) the 1st and (b) the 2nd exothermic peaks of GSPZ-EP.

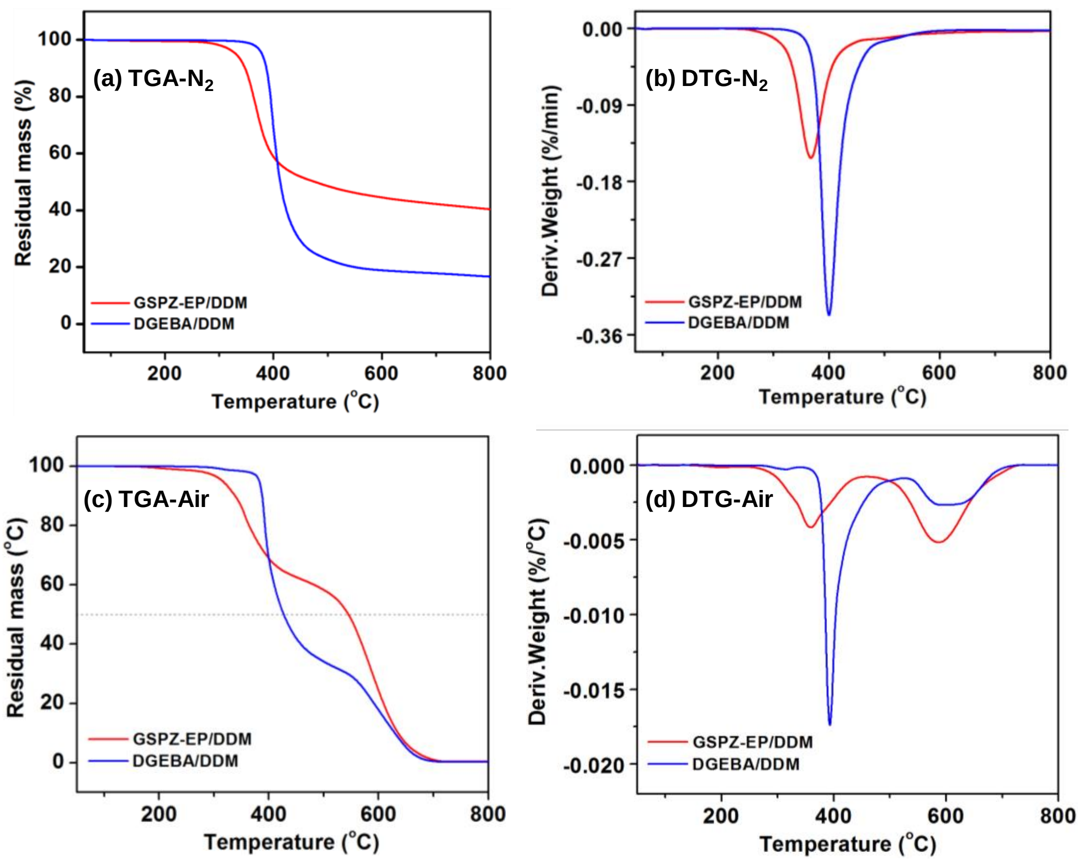

Supplementary Figure 10 Thermal stability of the cured resins. (a) TGA and (b) DTG curves of cured GSPZ-EP/DDM and DGEBA/DDM with heating rate of 20 K·min^{-1} under a nitrogen atmosphere; (c) TGA and (d) DTG curves of cured GSPZ-EP/DDM and DGEBA/DDM with heating rate of 20K·min^{-1} under an air atmosphere.

Supplementary Note 1

Kinssinger equation.

$$\ln\left(\frac{\beta}{T_p^2}\right)=\ln\left(\frac{AR}{E_a}\right)-\frac{E_a}{RT_p}$$

Where, β is the heating rates, T_p is the peak temperature, E_a is the apparent activation energy of the curing reaction, A is the pre-exponential factor, and R is the universal constant (8.314 $J \cdot mol^{-1} \cdot k^{-1}$).

Supplementary Note 2

The detailed explanation of Starink equation.

$$\ln\left(\frac{\beta_i}{T_{\alpha,i}^{1.92}}\right)=Const\text{-}1.0008\left(\frac{E_a}{RT_a}\right)$$

Where, β_i is the heating rate, $T_{\alpha,i}$ is the temperature at an equivalent conversion at various heating rates. E_α is the activation energy for a given conversion, and R is the universal constant (8.314 $J \cdot K^{-1} \cdot mol^{-1}$).

Starink method was used here to calculate the activation energy for different conversion. Based on the Starink equation, $\ln(\beta/T_\alpha^{1.92})$ vs. $1/T_p \times 1000$ values for the same fractional extent of conversion for a serious of dynamic DSC experiments carried out at various heating rates can be plotted to afford a straight line whose gradient corresponds to $-1.0008E_a/R$. Supplementary Figure 9 shows the starink plots for the 1st and 2ed exothermic peaks of GSPZ-EP in the conversion of 0.1-0.9, with a step of 0.1, and both the two exothermic peaks show the good linear relationship.
